# NO and H_2_S Contribute to Crop Resilience against Atmospheric Stressors

**DOI:** 10.3390/ijms25063509

**Published:** 2024-03-20

**Authors:** Francisco J. Corpas

**Affiliations:** Group of Antioxidants, Free Radicals and Nitric Oxide in Biotechnology, Food and Agriculture, Department of Stress, Development and Signaling in Plants, Estación Experimental del Zaidín, Spanish National Research Council (CSIC), Profesor Albareda 1, E-18008 Granada, Spain; javier.corpas@eez.csic.es

**Keywords:** acid rain, abiotic stress, gasotransmitters, oxidative stress, ozone, persulfidation, posttranslational modifications, *S*-nitrosation

## Abstract

Atmospheric stressors include a variety of pollutant gases such as CO_2_, nitrous oxide (NOx), and sulfurous compounds which could have a natural origin or be generated by uncontrolled human activity. Nevertheless, other atmospheric elements including high and low temperatures, ozone (O_3_), UV-B radiation, or acid rain among others can affect, at different levels, a large number of plant species, particularly those of agronomic interest. Paradoxically, both nitric oxide (NO) and hydrogen sulfide (H_2_S), until recently were considered toxic since they are part of the polluting gases; however, at present, these molecules are part of the mechanism of response to multiple stresses since they exert signaling functions which usually have an associated stimulation of the enzymatic and non-enzymatic antioxidant systems. At present, these gasotransmitters are considered essential components of the defense against a wide range of environmental stresses including atmospheric ones. This review aims to provide an updated vision of the endogenous metabolism of NO and H_2_S in plant cells and to deepen how the exogenous application of these compounds can contribute to crop resilience, particularly, against atmospheric stressors stimulating antioxidant systems.

## 1. Introduction

Higher plants, as sessile organisms, are recurrently subjected to environmental changes throughout their life cycle. Among the different atmospheric stressors, it can be mentioned that high and low temperatures, hailstorms, absence of rain (drought), extreme rain (waterlogging), ozone, ultraviolet (UV-B) radiation, CO_2_, methane, or nitrogen oxide (NOx) among others which effects on plants can be increased under the current climate change pattern [1,2,3]. The majority of them have a natural origin, but the negative effects of some of them could be increased by human activity. Furthermore, these atmospheric pollutants can affect extensive areas, but others can affect more restricted areas due to local phenomena, for example, the emissions of polluting gases by volcanoes or certain industries. However, the degree of pollution effects on a specific plant will depend on its intensity and the distance from the emission source.

**Figure 1:**
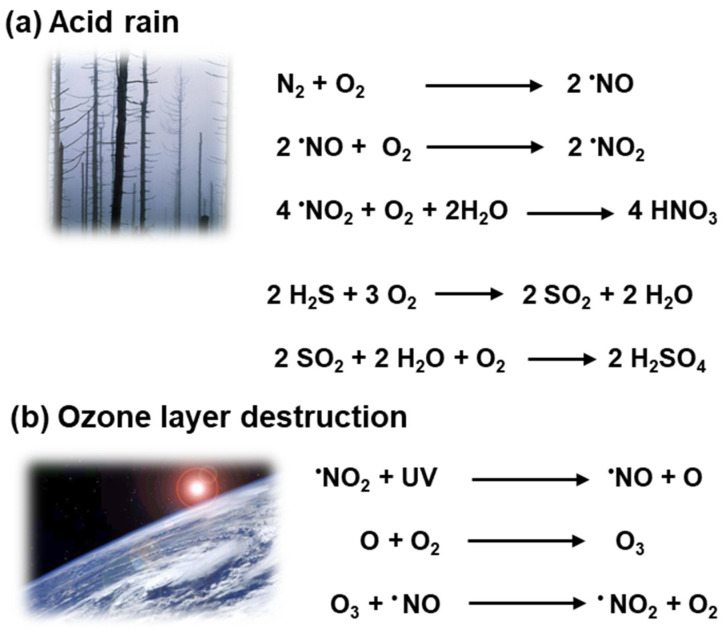
Nitric oxide (^•^NO) and hydrogen sulfide (H_2_S) participate in atmospheric pollution such as acid rain and the destruction of the ozone layer. (**a**) Nitrogen (N_2_) has a greater presence in the atmosphere but in the occurrence of atmospheric oxygen, it quickly transforms into nitrogen dioxide (^•^NO_2_). Nitrogen oxides are acidic, and they can form nitric acid (HNO_3_) which can be dissolved in water, giving rise to acid rain. Similarly, H_2_S can also react with O_2_ to generate sulfur dioxide (SO_2_) which reacts with water droplets in clouds to create sulfuric acid (H_2_SO_4_). (**b**) ^•^NO_2_ due to ultraviolet (UV) radiation generates ^•^NO and atomic oxygen, which together with O_2_ generates ozone, which reacts with ^•^NO, generating ^•^NO_2_ and oxygen, which constitutes the photolytic cycle of the destruction of the O_3_ layer.

Nitric oxide (^•^NO) is a free radical that is part of the nitrogen cycle and in the atmosphere, in the presence of oxygen, it quickly transforms into nitrogen dioxide (^•^NO_2_), and both constitute nitrogen oxide (NOx). Figure 1a,b illustrates how atmospheric ^•^NO, as a pollutant, participates in the formation of acid rain as well as in the destruction of the ozone layer [4,5]. For many plant species, the negative effects triggered by nitrogen oxides (NOx) have been estimated when the level of NOx is around 30 µg/m^3^. However, there is experimental evidence suggesting that moderate concentrations of NOx may have both positive and negative plant growth responses [6,7].

On the other hand, atmospheric hydrogen sulfide (H_2_S) comes from different sources such as volcanoes, geothermal vents, or wetlands where it is generated by bacteria during the anaerobic decay of organic sulfur compounds [8,9,10]. In the atmosphere, H_2_S is oxidized to sulfur dioxide (SO_2_), which then can be converted to sulfuric acid (H_2_SO_4_) and participates in acid rain (Figure 1a).

From the time when ^•^NO and H_2_S were identified and characterized in the 18th century, these molecules have been considered toxic molecules that exert negative effects on all organisms. At the end of the 20th century, it was found that ^•^NO and H_2_S can be generated endogenously in both animal and plant cells [11,12,13,14]. As a result, the concept of “toxic” molecules changed, and to date, they have been shown to both exert regulatory and signaling functions in many plant processes such as seed germination, root development, plant growth, stomatal movement, senescence, fruit development and ripening as well as response mechanisms to both abiotic and biotic stresses [15,16,17]. Thus, both ^•^NO and H_2_S have paradoxical effects as atmospheric pollutants but also as signaling molecules that are endogenously generated in cells. Likewise, there are numerous examples that their exogenous application, individually or in combination, exerts beneficial effects against atmospheric stress.

This review aims to provide an updated vision of the endogenous metabolism of ^•^NO and H_2_S in plant cells and to deepen how the exogenous application of these compounds can contribute to crop resilience against some representative atmospheric stressors such as extreme temperature, O_3_, UV-B radiation, and acid rain.

## 2. ^•^NO and H_2_S Metabolism in Higher Plants

Our knowledge about ^•^NO and H_2_S metabolism has increased significantly during the last decade considering that these two molecules were considered toxic until they were found to be endogenously generated in animal cells [13,14].

The enzymatic generation of ^•^NO in higher plants has been very controversial since its generation was discovered. Currently, two main enzymatic pathways have been generally accepted, the reductive and the oxidative pathways [18,19,20]. The reductive pathway is the one that uses nitrate and nitrite as substrates using NADH as an electron donor, being linked to the nitrate reductase (NR) and nitrite reductase (NiR) activities [21,22,23,24]. On the other hand, there is the oxidative pathway, which is considered similar to the nitric oxide synthase (NOS) of animal cells, since it starts with L-arginine using NADPH as the electron donor and FAD, FMN, calcium, calmodulin, and tetrabiopterin as cofactors, so it is called L-Arg-dependent NOS-like activity because the gene similar to that of animal organisms that encodes it has not been identified [25,26,27]. In addition, there is another possible route that, from polyamines or oximes, seems to be involved in the generation of ^•^NO [28,29,30]. However, we must not rule out other possible enzymatic or non-enzymatic sources that should be involved in the generation of ^•^NO.

**Figure 2:**
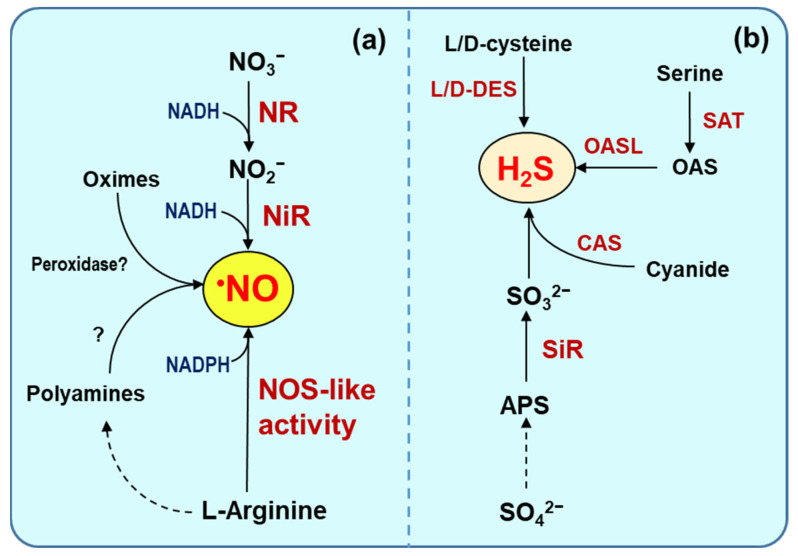
Main enzymatic source of ^•^NO and H_2_S in higher plant cells. (**a**) Nitrate reductase (NR), nitrite reductase (NiR), and L-arginine-dependent nitric oxide synthase (NOS)-like activity are the recognized major candidates for enzymatic ^•^NO sources in the different subcellular compartments of higher plants. (**b**) The biosynthesis of H_2_S in plants is part of sulfur and cysteine metabolism which primarily involves several enzymes located in the cytosol, plastids, and mitochondria including L/D-cysteine desulfhydrase (L/D-DES), cyanoalanine synthase (CAS), serine acetyltransferase (SAT), sulfite reductase (SiR), and O-acetyl-l-serine(thiol)lyase (OASL), also named cysteine synthase. APS, adenosine 5′-phosphosulfate. Dashed line, indicates different stages. ?, unidentified.

The generation of H_2_S in plants is part of the sulfate assimilation pathway and the cysteine biosynthesis pathway. Currently, there are several enzymes located in different subcellular compartments involved in the generation of H_2_S [31,32]. Figure 2a,b shows the main enzymatic source involved in the generation of ^•^NO and H_2_S in higher plants.

## 3. ^•^NO- and H_2_S-Derived Posttranslational Modifications (PTMs) as Tools to Regulate Plant Metabolism

^•^NO and derived molecules called reactive nitrogen species (RNS) can affect the function of different macromolecules through their specific interactions. Among the RNS, it is worth highlighting peroxynitrite (ONOO^−^) which is the result of the chemical reaction between ^•^NO and superoxide radical (O_2_^•−^) [33] or S-nitrosoglutathione (GSNO), which results from the interaction of ^•^NO with reduced glutathione (GSH) [34,35]. RNS can mediate several post-translational modifications (PTMs) that affect different macromolecules including peptides, proteins, fatty acids, and nucleotides. Thus, RNS interacts with thiol groups present in Cys residues to generate the corresponding *S*-nitrosated protein, with tyrosine residues to generate tyrosine nitration or bind to metals present in certain proteins in a process designed as metal nitrosylation [36,37,38,39]. ^•^NO can also interact with other biomolecules including unsaturated fatty acids (FAs) to form the corresponding nitro-FAs [40] and nucleic acids through guanine or guanosine to generate 8-nitroguanine or 8-nitroguanosine, respectively [41,42].

**Figure 3:**
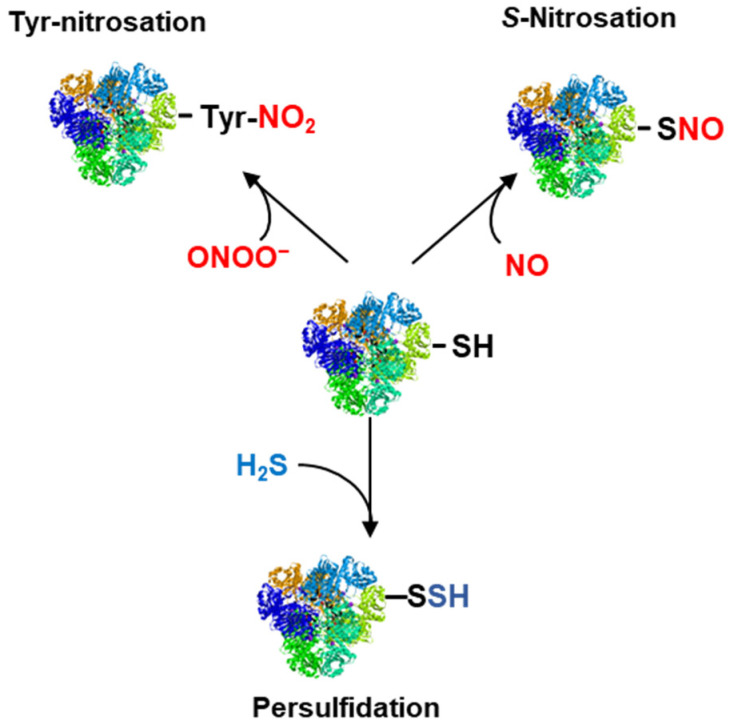
Protein postranslational modifications (PTMs) mediated by either ^•^NO (*S*-nitrosation and tyrosine nitration) or H_2_S (persulfidation). ONOO^−^, peroxynitrite.

H_2_S mediates another PTM named persulfidation which involves its interaction with the thiol group (-SH) of susceptible Cys residues. Similar to *S*-nitrosation, persulfidation is a reversible covalent interaction but, in this case, the thiol group is converted into a persulfide (-SSH) group which can affect positively or negatively the function of the target proteins [43,44]. Figure 3 illustrates the main PTMs mediated by ^•^NO and H_2_S. However, in a cellular context, it should be considered that the thiol groups of Cys residues are susceptible to being targets of other thiol-based oxidative posttranslational modifications (OxiPTMs) mediated by glutathione (*S*-glutathionylation), H_2_O_2_ (*S*-sulfenylation), fatty acids (*S*-acylation) or cyanide (*S*-cyanylation) that can compete with each other depending on their cellular concentrations and the subcellular location of the target protein [45,46,47,48,49]. However, in conditions of oxidative stress resulting from environmental stress, some of them may have a greater preponderance, such as an increase in H_2_O_2_.

## 4. Stomata Movement, a Process Regulated by ^•^NO and H_2_S

**Figure 4:**
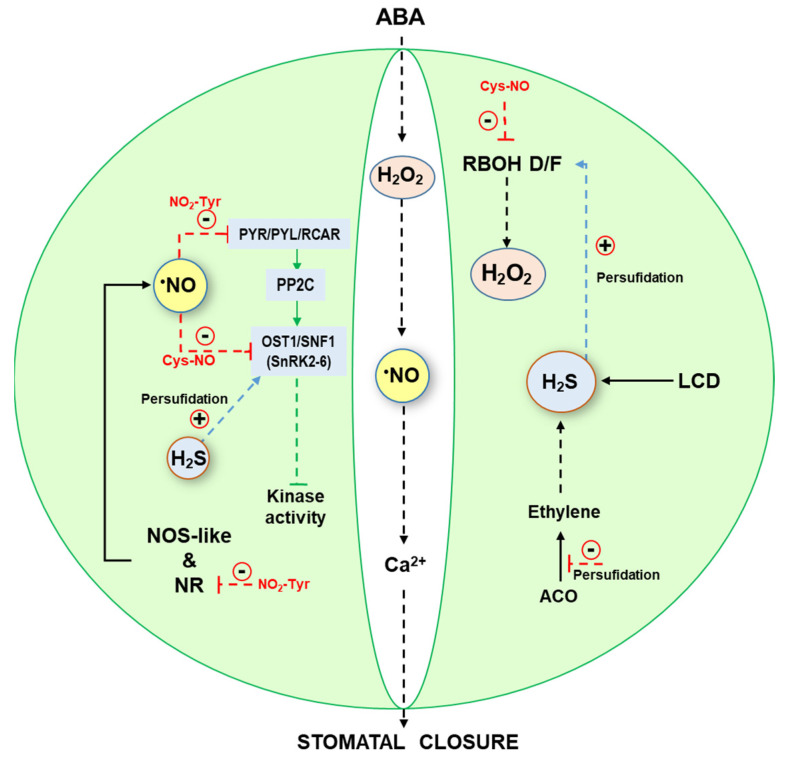
Simple model of the signaling cascade mediated by abscisic acid (ABA), H_2_O_2_, ethylene and Ca^2+^ where ^•^NO and H_2_S participate in the stomatal closure in response to atmospheric stresses. PP2C, protein phosphatase 2C; PYR/PYL/RCAR, pyrabactin resistance1/PYR1-like/regulatory components of ABA receptor. Red dashed lines indicate inhibitory effects. Blue dashed arrows indicate positive effects. Green dashed line, indicates blocking of activity.

Stomata are specialized cells that regulate gas exchange in the leaves and stomatal closure is one of the response mechanisms against atmospheric stress [50,51,52]. It is interesting to mention that both ^•^NO and H_2_S are molecules that, although they may be polluting molecules, are also generated endogenously by regulating stomatal closure through PTMs including tyrosine nitration, *S*-nitrosation, and persulfidation. Thus, ^•^NO and H_2_S are part of the crosstalk with other signal molecules such as abscisic acid (ABA), Ca^2+^, H_2_O_2_, and ethylene among others participate in the regulation of stomatal movement [53,54,55,56,57,58]. Figure 4 shows a simple model of the main signals involved in the stomata closure where it highlights the main effect of ^•^NO and H_2_S. Thus, ^•^NO seems to be generated either via NR or a NOS-like activity whereas H_2_S is generated by an L-cysteine desulfhydrase (LCD) activity. NR can be inhibited by tyrosine nitration (NO_2_-Tyr) [24]. On the other hand, H_2_O_2_ is produced by a respiratory burst oxidase homolog (RBOH) type D/F. H_2_S triggers the generation of H_2_O_2_ by persulfidation of RBOH [59] whereas it can be inhibited by *S*-nitrosation.^•^NO can inactivate the ABA receptor PYR/PYL/RCAR by a process of tyrosine nitration (NO_2_-Tyr), but ^•^NO can also negatively regulate the open stomata 1 (OST1)/sucrose nonfermenting 1 (SNF1)-related protein kinase 2.6 (SnRK2.6) complex by *S*-nitrosation (Cys-NO). But SnRK2.6 can be activated by persulfidation [60,61]. On the other hand, ethylene induces H_2_S production in guard cells and this H_2_S can then inhibit the synthesis of ethylene by the inhibition of 1-aminocyclopropane-1-carboxylic acid oxidase (ACO) activity by persulfidation (Cys-SSH) at Cys60 [62]. 

Thus, it is well established that stomata movement as it has happened with photosynthesis activity can be affected by numerous atmospheric pollutants [63,64,65,66,67,68].

## 5. Atmospheric Pollutants and Higher Plant Response—What Happens to ^•^NO and H_2_S When It Is Applied Exogenously?

At present, it is known that plants can emit ^•^NO [11,69,70,71] and H_2_S [12,72,73] to their surrounding atmosphere; however, plants could also release other gases such as CO_2_, nitrous oxide (N_2_O) [74,75] and methane (CH_4_) [76,77] which are part of the greenhouse gases that contribute to global warming. At the same time, it is important to note that atmospheric ^•^NO/NOx and H_2_S may be adsorbed at the leaf’s surface through the stomata [65,78,79,80], and depending on their concentration, these gases can have either negative or beneficial effects on higher plants. For example, it has been pointed out that ^•^NO seems to be a key signaling molecule in the mechanism of response against higher levels of atmospheric gases including CO_2_, N_2_O, CH_4_, or O_3_ which usually provoke stress in plants that have associated oxidative stress because they trigger an uncontrolled increase in the generation of ROS and RNS associated with a lower antioxidant capacity [81]. Thus, the harmful or beneficial effects of the gas exchanges between plants and the surrounding atmosphere will depend on their final concentration inside the cells.

On the other hand, ^•^NO and H_2_S as signaling molecules that are involved in numerous biological processes in higher plants, have started to be applied exogenously as alternative biotechnology tools since it has been proven that they can exert benefit effects to palliate the negative effects caused by different atmospheric factors such as high and low temperatures, O_3_, UV-B radiation or acid rain among others.

### 5.1. High and Low Temperature

Higher plants, during their development, are exposed to seasonal changes in temperature; consequently, they have developed the corresponding strategic adaptations that have allowed them to survive in a specific ecosystem [82,83,84]. However, plants can also undergo unusual extreme temperatures provoking undesirable effects. For example, *Arabidopsis thaliana* exposed to heat stress (38 °C) experiences an increase in the H_2_O_2_ content in chloroplasts which triggers the *S*-sulfenylation of the 2-phosphoglycolate phosphatase 1 at Cys86 producing its inhibition and, consequently, provoking the accumulation of 2-phosphoglycolate which has toxic effects because it inhibits the enzymes triose-phosphate isomerase and phosphofructokinase which are required for CO_2_ assimilation [85]. In these cases, plants have to trigger a different mechanism of responses in which ^•^NO and H_2_S, along with other regulatory molecules, participate to react and alleviate possible damages caused by extreme temperatures [86,87,88,89,90,91].

Table 1 and Table 2 show some examples of how ^•^NO and H_2_S applied exogenously can contribute to reducing the damage associated with high and low temperatures and how antioxidant systems are stimulated to alleviate oxidative damages associated with extreme temperatures. It should be mentioned that in the majority of studies in plants, the most widely used donors are sodium nitroprusside (SNP) for ^•^NO and sodium hydrosulfide (NaHS) for H_2_S. The main reason is that both donors have a low economic cost compared to other ^•^NO donors such as GSNO or NONOates or H_2_S donors such as GYY4137 or sulfobiotic-H_2_S donors 5a, 8ℓ, and 8o. SNP and NaHS donors are usually applied either by spraying the aerial part of the plant or by adding it to the nutrient solution.

### 5.2. Ozone (O_3_)

According to the predictions of Wang et al. [92], the increase in atmospheric O_3_ has been estimated to be 20–25% by 2050 and it has already been proven that a high content of O_3_ can negatively affect plant metabolism and growth [93,94,95] which usually triggers an increase in ROS metabolism [96,97]. For example, in tobacco plants exposed to O_3_, an accumulation of ^•^NO and H_2_O_2_ was found [98]. In the case of *Phaseolus vulgaris*, O_3_ reduces the chlorophyll content and increases the content of ROS [99]. Table 1 and Table 2 show some representative examples of how ^•^NO and H_2_S applied exogenously to plants can contribute to providing metabolic adaptations to high levels of atmospheric O_3_.

### 5.3. UV-B Radiation

UV radiation is a non-ionizing radiation that is produced by the sun and three categories of UV radiation can be distinguished according to the wavelength: 315–400 nm corresponds to UV-A, 280–315 nm to UV-B, and 100–280 nm to UV-C. UV-B radiation is the most studied in plants due to its increase on the earth’s surface as a consequence of the depletion of the stratospheric O_3_ layer since the atmosphere intercepts around 77% UV radiation. In this sense, plants under UV-B radiation trigger nonspecific responses such as DNA damage and an increase in ROS production as well as specific ones that involve photomorphogenic signals affecting the gene expression of *UV-resistance locus 8* (*UVR8*) and *constitutive photomorphogenesis 1* (*COP1*) accompanied by the transcription factor elongated hypocotyl 5 (HY5) [100,101,102,103,104].

Accumulating data indicate that in plants under UV-B radiation, the metabolism of ^•^NO and H_2_S is exacerbated and contributes to palliating the damaged symptoms [105,106,107,108,109,110]. For example, in leaves of kidney beans (*Phaseolus vulgaris*) exposed to UV-B stress it was found that ^•^NO generation was associated with a NOS-like activity being mediated by H_2_O_2_ [106]. Additionally, the exogenous application of ^•^NO and H_2_S has been shown to contribute at different levels to diminishing the negative impact of UV-B radiation mainly by stimulating at gene and protein levels the different antioxidant systems. Table 1 and Table 2 display representative examples of how exogenous ^•^NO and H_2_S applied can palliate the negative impact of UV-B radiation in plants.

### 5.4. Acid Rain

As mentioned above, acid rain is the consequence of the presence of NOx and/or SO_2_ in the air during precipitation (Figure 1a). Acid rain damages plant growth since it affects photosynthesis and, in general, triggers a response of the antioxidant systems to palliate the oxidative stress [5,111,112]. Some examples show that the exogenous application of several compounds such as glutathione, melatonin, or silicon could help to palliate the harmful effect on plants [113]. In the model plant, *Arabidopsis thaliana* exposed to acid rain has been found to have an active nitrogen metabolism which has an elevated ^•^NO production and provides a tolerance to acid rain [111]. Table 1 summarizes how the exogenous ^•^NO application modulates the plant response to acid rain.

**Table 1:** Main effects of the exogenous application of ^•^NO plants exposed to diverse atmospheric stressors.

^•^NO Donor	Plant Species	Effects	Ref.
**Low temperature**			
0.1 mM SNP	Jujube (*Ziziphus jujube*) fruit	Exogenous NO inhibits the development of chilling injury by maintaining cellular redox homeostasis through the presence of *S*-nitrosation of superoxide dismutase and catalase.	[114]
0.2 mM SNP	Cowpea (*Vigna unguiculata*)	Diminish the production of ROS and the content of MDA. Delay the degradation of photosynthetic pigments, increase the content of proline, and the activity of antioxidant enzymes such as SOD, catalase, and component of the ascorbate-glutathione cycle.	[115]
50 µM GSNO	Chinese Cabbage (*Brassica rapa*)	Simultaneous ^•^NO treatment with brassinosteroids increases the leaf area, stem diameter, chlorophyll content, dry and fresh weight, and proline content. Decrease the MDA content.	[116]
**High temperature**			
50 µM SNP	Wheat (*Triticum aestivum* L.)	Improve growth and photosynthetic parameters. Mitigate the oxidative stress. Increase membrane stability index.	[117,118]
100 µM SNP	Rice (*Oryza sativa* L.)	^•^NO interacts with ethylene and H_2_S metabolism. Activation of the antioxidant system such as components of the ascorbate–glutathione cycle, accumulation of osmolytes with the concomitant increase in thermos tolerance.	[119,120]
**Ozone (O_3_)**			
50 µM SNP	*Arabidopsis thaliana*	^•^NO enhances O_3_-induced cell death, possibly by altering the NO–ROS balance. Decrease salicylic acid and increase jasmonic acid concentrations.	[121]
200 μM SNP	Wheat (*Triticum aestivum* L.)	NO is involved in ozone tolerance. It enhances the net photosynthetic rate while reducing H_2_O_2_, membrane peroxidation, and electrolyte leakage. Increase SOD and POD activities.	[122]
**UV-B radiation**			
SNP	Bean (*Phaseolus vulgaris*) leaves	Decrease chlorophyll contents and oxidative damage to the thylakoid membrane. Increase activities of SOD, APX, and catalase.	[123]
100 µM SNP	Maize (*Zea mays* L.) leaves	Induce the accumulation of flavonoids and anthocyanin that absorb UV-B radiation.	[124]
0.8 mM SNP	Soybean leaves (*Glycine max* L.)	Up-regulate the gene expression and activity of antioxidant enzymes	[125]
**Acid rain**			
0.5 mM SNP	Longan (*Dimocarpus longana*) seedlings	Under acid rain (pH 3.0), exogenous ^•^NO provokes an increase in total chlorophyll, soluble protein, and soluble sugar as well as the activity of antioxidant enzymes (SOD, POD and CAT) whereas it decreases the MDA content.	[126]
0.1 mM SNP	*Arabidopsis thaliana*	^•^NO treatment decreases the leaf necrosis whereas it increases the fresh weight.	[111]
0.25 mM SNP	*Vigna radiata* seedlings	Under simulated acid rain (pH 2), exogenous ^•^NO triggers an increase in antioxidant activities (SOD, POD, and APX), NR activiy and NO content whereas it decreases MDA content.	[7]

APX, ascorbate peroxidase. CAT, catalase. GSNO, S-nitrosoglutathione. MDA, malondialdehyde. NR, nitrtate reductase. POD, peroxidase. SNP, sodium nitroprusside. SOD, superoxide dismutase.

**Table 2:** Main effects of the exogenous application of H2S plants exposed to diverse environmental stressors.

H_2_S Donor	Plant Species	Effects	Ref.
**Low Temperature**			
50 μM NaHS	Cucumber (*Cucumis sativus* L.)	Increase the content of GSH’ and cucurbitacin C. H_2_O_2_ as a downstream signal of IAA mediates H_2_S-induced chilling tolerance.	[127,128]
0.5 mM NaHS	Pepper (*Capsicum annuum* L.)	Increase the content of endogenous H_2_S and the integrity of the membrane system. Enhance the photosynthetic rate, stomatal conductance, transpiration rate, and photosynthesis. Reduce the intercellular CO_2_ concentration. Increase antioxidant activities (SOD, catalase, and ascorbate-glutathione cycle).	[129]
0.5 mM NaHS	Blueberry (*Vaccinium corymbosum*) leaves	Promote the electron transfer from Q A to Q B on the PSII acceptor side and alleviate the degradation of chlorophyll and carotenoids. Increase proline content.	[130]
0.5 mM NaHS	Alfalfa (*Medicago truncatula*)	Improve the height, number of leaves, and fresh and dry shoot weights. Increase tolerance by regulating the antioxidant defense system and enhancing photosynthetic capacity.	[131]
**High temperature**			
100 μM NaHS	Strawberry (*Fragaria* × *ananassa* cv. ‘Camarosa’)	Induction of gene expression of antioxidant enzymes (cAPX, CAT, MnSOD, GR), heat shock proteins (HSP70, HSP80, HSP90) and aquaporins (PIP).	[132]
500 μM NaHS	Maize (*Zea mays* L.)	Improve seed germination and increase antioxidant enzymes. Accumulation of proline.	[133]
50 μM NaHS or 10 μM GYY4137	Poplar (*Populus trichocarpa*)	Increase GSNOR activity and reduce HT-induced damage to the photosynthetic system.	[134]
100 μM NaHS or 10 μM GYY4137	*Arabidopsis thaliana*	Enhance seed germination rate under HT. Increase gene expression of *ABI5* (ABA-INSENSITIVE 5).	[135]
**UV B radiation**			
125 μM NaHS	Borage (*Borago officinalis* L.)	Decrease the MDA carbonyl groups, and H_2_O_2_ content. Increase the activities of APX and guaiacol peroxidase.	[136]

ABA, abscisic acid. APX, ascorbate peroxidase. CAT, catalase. GSH, reduced glutathione. IAA, indole-3-acetic acid. MDA, malondialdehyde. NaHS, sodium hydrosulfide. SOD, superoxide dismutase.

## 6. Conclusions and Future Perspectives

**Figure 5:**
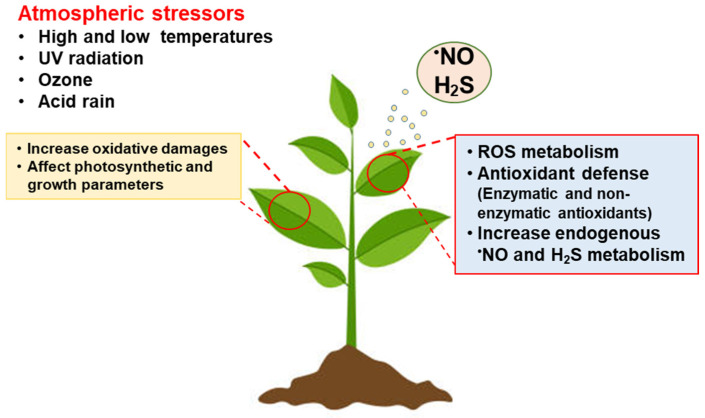
Working model of the main effects of the exogenous application of ^•^NO or H_2_S under several atmospheric stressors which trigger an active ROS metabolism with the induction of antioxidant systems.

^•^NO and H_2_S have become paradoxical molecules in plant biology since they have gone from being hazardous molecules to becoming essential molecules in cellular metabolism, regulating physiological processes from seed germination, root development, photosynthesis, senescence, stomatal closure, formation of flowers and fruit ripening, in addition to participating in the response mechanisms against challenging environments. Paradoxically, the available information demonstrates that the exogenous application of these molecules can be biotechnological tools that allow for promoting crop resilience [137,138]. In most cases, these gasotransmitters stimulate enzymatic and non-enzymatic antioxidant systems, for example, the APX activity is upregulated by S-nitrosation and persulfidation [139,140] which makes it possible to alleviate oxidative damage associated with atmospheric stressors, protecting the functionality of cells, and maintaining photosynthetic activity (Figure 5). Although we are still in the basic studies to understand the intimate molecular mechanisms exerted by ^•^NO and H_2_S, it would be of great interest to establish protocols on how the exogenous application of these molecules can allow us to combat atmospheric stressors or other types of abiotic or biotic stresses, allowing us to connect the basic knowledge and its application to the agricultural productive sector [141,142].
